# 

**Published:** 1877-06

**Authors:** 


### CONTENTS:

ARTICLE I.—Death from Nitrous Oxide Gas. By S. Parsons Shaw.............. 49

ARTICLE II.—Dental Association Meetings. South Carolina State Dental Association 52

Pennsylvania State Dental Society.............................. 53

American Dental Association.................................. 53

Tennessee Dental Association................................. 54

Southern Dental Association.................................. 54

ARTICLE III—The Discovery of Anæsthesia. By J. Marion Sims, M. D. ........ 55

ARTICLE IV.—Nitrous Oxide as an Anæsthetic By H. Gibbons, M. D........... 69

ARTICLE V.—Sub-Luxation of Inferior Maxilla Backwards. By E. N. Moore, Jr. M. D. 75

ARTICLE VI.—Discoloration of Gold Plugs.................................. 78

ARTICLE VII.—Radical Cure of Neuralgia of the Inferior Dental Nerve.......... 81

ARTICLE VIII.—Anomalies in Digestion...................................... 83

ARTICLE IX.—A Simple Cautery....... ..... ............... ............ 85

ARTICLE X.—A New Dental Inhaler........................................ 87

ARTICLE XI.—Croton Chloral in Dentistry. By C. J. Cleborne, M. D........... 87

### EDITORIAL, ETC.

Death from Nitrous Oxide Gas and Ether.......................................... 89
Solvent for Bubber................................................ 91
Symptoms Resulting from Anæsthesia by Ether in Young Subjects ............... 91
Indigestible Substances................................................ 91
Acquittal of Dr. Warren C. Westlake............................................... 92

### MONTHLY SUMMARY.

Local Action of Chloral Hydrate.................................................. 92

Carbolized Camphor and its Therapeutic Applications.......................... 93

Rubber vs., Celluloid.................................................. 94

Preliminary Education of Medical Students.......................................... 95

Blue Glass.................................................................. 95

Advice to Students...... .................................................... 96

Stimulants used by the Race .................................................. 96

An Iron Calculus.................................................................. 96

